# Numerical analysis of photonic crystal fiber of ultra-high birefringence and high nonlinearity

**DOI:** 10.1038/s41598-020-77114-x

**Published:** 2020-12-03

**Authors:** Patrick Atsu Agbemabiese, Emmanuel Kofi Akowuah

**Affiliations:** Department of Computer Eng, Kwame Nkrumah University of Science and Technology, Kumasi, Ghana

**Keywords:** Optical materials and structures, Other photonics

## Abstract

A numerical analysis of a hexagonal PCF structure with four circular air hole rings around the core has been presented in this paper. By utilizing a full vectorial finite element method with perfectly matched layers, propagation properties such as birefringence, chromatic dispersion and confinement losses are numericaly evaluated for the proposed PCF structure. Specifically, birefringence of 2.018 × 10^–2^, nonlinear coefficients of 40.682 W^−1^ km^−1^, negative chromatic dispersion of − 47.72 ps/km.nm at 1.55 µm and − 21 to − 105 ps/km.nm at the telecommunication band of C-U have been reported.

## Introduction

Photonic crystal fibers (PCFs) have outperformed its conventional step index fiber counterpart. This is due to the flexibility in the design of the structure, the material used and application. PCFs referred to as microstructures or holey fibers have received growing interest in recent years. They are classified into two categories according to its light guiding mechanism; photonic band gap guiding and refractive index guiding which is based on total internal reflection. Both band gap and index guiding PCFs have claddings which consist of air holes, although they differ in the structure of the core^1,2^. In Solid core PCfs, the refractive index contrast is achieved by the arrangement of air holes in the cladding^3^. PCFs have a range of degrees of freedom and design flexibility to control optical properties such as endless single mode^4^, high birefringence^5^, chromatic dispersion management^6^, large mode area^7^, high nonlinearity^8^ and low confinement loss^9^. These properties open the door for a lot of applications like; nonlinear optics^10^, sensing^1^, high power technology^11^ and telecommunications^12,13^. The optical properties can be achieved through optimisation of air hole shape, size and position^14–17^.

Birefringence is generally categorized into two; these are geometrical birefringence and stress birefringence^18,19^. Optical fibers are doped in order to increase the refractive index, change the melting properties and induce birefringence^1^. High Birefringence is required to keep two linear orthogonal polarisation states over long distance. High birefringent PCFs are highly sensitive to temperature, a characteristic which is important in sensing applications^20^.

Dispersion is a measure of the temporal broadening of the optical pulse and it is one of the main sources of penalty for efficient transmission of optical signals. A PCF of high Birefringence and negative dispersion compensation is important in optical amplification as it maintains linear polarisation and useful for double Raman gains and sensing^21^. Furthermore, research has shown that achieving high birefringence, high nonlinearity and negative dispersion can be useful for polarization control in fiber optic sensors, telecommunication applications and broadband dispersion compensation in high-bit-rate transmission systems^22^. A lot of work has been reported recently to achieve high birefringence, high nonlinearity and dispersion management through the use of elliptical holes around the core^23,24^, using different shapes of air holes other than circular in or around the core^25–27^, defective core^21^, doping the core with gas or liquid and hybrid cladding^28,29^, double cladding^22^, dual core photonic bandgap^30^, triangular lattice^31^ and other shapes of air hole rings apart from the widely used hexagonal lattice^32–35^. Rhombic and elliptical hole PCFs have been presented^26^ to achieve birefringence (B) of 8 × 10^–3^ and Confinement loss(CL) of 5 × 10^–4^ dB/m at 1.55 µm. However chromatic dispersion, which plays a key role in optical communication was not reported on by^26^. Furthermore, fabrication of such structures are relatively difficult considering the complex designs rhombic desings present^26^. There are also reports of PCF structures combining circular and elliptical holes to achieve^36^ B of 10^–3^ and a CL of 10^−7^ dB/m. Despite the impressive performance of the structures presented by^36^, the chromatic dispersion properties are not presented by the authors^36^. Similar to the strutctures presented by^26^, these structures are bulky, complex and ccostly to fabricate^36^.

There is a growing interest in hybrid PCF structures which employ two or more lattice structures. One such example is presented by^28^, which consists of hexagonal and decagonal structures with five air hole rings. The structure reported CL of 8.13 × 10^−3^ dB/m and B of 2.6 × 10^–2^ with a very high negative compensation at 1.55 µm, which is similar to^37^. In the PCF structure by^5,38^ a very high B of 10^–3^ at a low CL have was reported but elliptical air holes incorporated in the core of the structures make fabrication difficult. A design with seven rectangular air holes located in the core has been presented in^27^ with very low loss. However the fabrication of rectangular holes would be difficult. Paul et al.^23^, designed a PCF of square lattice with five air hole rings made up of both circular and elliptical air holes. A very high value of dispersion compensation (DC) and a high B of 4.74 × 10^–3^ has been achieved however, owing to the number of elliptical holes present in the design, the fabrication of the structure would be difficult.

Another high B in the order of 10^–2^ and very large negative dispersion is presented in^24,39,40^ but, the structure contains elliptical holes around the core which increases fabrication difficulty. A dual concentrated core with five layer air hole ring has been presented with high DC and low CL of 10^−4^ dB/m but no birefringence results were shown. In^21^, seven rings of square lattice structure of circular air holes was proposed. A high B of the order of 10^–2^ was obtained with negative chromatic dispersion. Furthermore, Arif et al. ^5^ demonstrated a nonlinearity of 39.330 W^−1^ km^−1^ but with a low B of 2.83 × 10^–3^. In^22^, high B of 3.12 × 10^–2^, nonlinearity of 24.89 W^−1^ km^−1^ and high negative dispersion has been proposed however the nonlinearity is low and the structure is complex and fabrication would be a challenge. It has also been demonstrated in^41^ that nonlinearity of 26.67 W^−1^ km^−1^ can be achieved at a pump wavelength of 1.3 µm. Bored core PCF reported in^42^, achieved a nonlinearity of 118.4 W^−1^ km^-−1^ and a negative dispersion value of − 2221 ps/km.nm but the birefringence (B) was not discussed.

It is clear from the aforementioned research works that it is very difficult to simultaneously achieve excellent birefringence, chromatic dispersion and nonlinear coefficient for a given PCF structure. This paper presents a proposed PCF structure with ultra-high B of 2.018 × 10^–2^ and nonlinear coefficients of 40.72w^−1^ km^−1^ at 1.55 µm. Chromatic dispersion of − 47.7 ps/km.nm at 1.55 µm has been demonstrated.

Our proposed PCF structure with high B and high nonlinearity within optical communication wavelengths is very desirable in high bit rate data communication, polarization maintaining and sensing applications.

## Design methodology

Refractive index of pure silica is dependent on the wavelength, and for this structure, SellMeier equation^43^ used:

1 $$n^{2} = 1 + \frac{{{\rm B}_{1} \lambda^{2} }}{{\lambda^{2} - c_{1} }} + \frac{{{\rm B}_{2} \lambda^{2} }}{{\lambda^{2} - c_{2} }} + \frac{{{\rm B}_{3} \lambda^{2} }}{{\lambda^{2} - c_{3} }}$$

where n is the refractive index of the silica and λ is the wavelength in µm. B_1,2,3_ and C_1,2,3_ are SellMeier coefficients as shown in Table 1.

**Table 1:** Values of sellmeier coefficients for background silica material.

Parameters	Constants
B1	0.69675
B2	0.408218
B3	0.890815
C1	4.67914826e-3
C2	1.35120631e-2
C3	97.9340025

In this paper in order to determine the optical properties of the PCF the cross section is segregated into subspaces where Maxwell equation are computed by determining the adjacent subspaces. The vectorial Eq. (2) is determined using anisotropic PML^14,44^:

2 $$\nabla \times \left( {\left[ {S^{ - 1} } \right]\nabla \times E} \right) - k_{0}^{2} n^{2} \left[ S \right]E = 0$$

where ko = 2π/λ is the wave number in vacuum, λ is the wavelength, E is the electric field vector, n is the refractive index of the domain, [s] is the PML matrix, [s]^−1^ is an inverse matrix of [s].

Dispersion is a key factor that reduces the information that the fiber cable can carry. The presence of dispersion results in the spreading of the pulse creating intersymbol interference. Dispersion can categorised into two ,that is intermodal and intramodal dispersion. Intermodal occurs in multimode fibers while intermodal occurs in single mode fibers. Dispersion is made up of the material dispersion and waveguide dispersion The chromatic dispersion is calculated with the formula^1^;

3 $$D = - \frac{\lambda }{c}\frac{{\partial^{2} Re(n_{eff} )}}{{\partial \lambda^{2} }}$$

where c, is the speed of light in free space and Re is the real value of the effective refractive index.

Birefringence(B) is the difference in the refractive indices of the two polarisation modes. It is calculated as^45^;

4 $$B = |n_{x} - n_{y} |$$

where *n*_*x*_ and *n*_*y*_ are the effective refractive indices for *x* and *y* polarization modes respectively.

The confinement loss is calculated from the imaginary part of the effective refractive index.

In PCFs the light propating through the core is due to the finite rings of air holes in the bulk silica which extends to infinity. Leakage of light from the core to the exterior matrix is unavoidable even though the jacket is far from the cladding and core region. This leakage of light causes confinement loses.The formula that is used to compute confinement loss is given by^44^

5 $$C_{loss} = \frac{40\pi }{{ln\left( {10} \right)\lambda }}{\text{Im}} \left( {n_{eff} } \right)\left[ \frac{dB}{m} \right]$$

where λ is the operating wavelength. I_m_(n_eff_) is the imaginary value of the effective refractive index.

The effective mode area is a parameter which determines the performance of the PCF and it is given by^14^:

6 $$A_{eff} = \frac{{\left( {\iint {\left| E \right|^{2} dxdy}} \right)^{2} }}{{\iint {\left| E \right|^{4} dxdy}}}$$

where E is the amplitude of the transverse electric field.

Non-linear coefficient (γ) determined in the core of the fiber is given by:

7 $$\gamma = \frac{2\pi }{\lambda }\frac{{n_{2} }}{{A_{eff} }}$$

where n_2_ is the refractive index coefficient and for this work 2.76 × 10^–20^ m^2^/W is used.

Beat length is calculated using;

8 $$L_{B} = \frac{\lambda }{{\left| {n_{x} - n_{y} } \right|}} = \frac{\lambda }{B}$$

## Simulation and results

**Figure 1:**
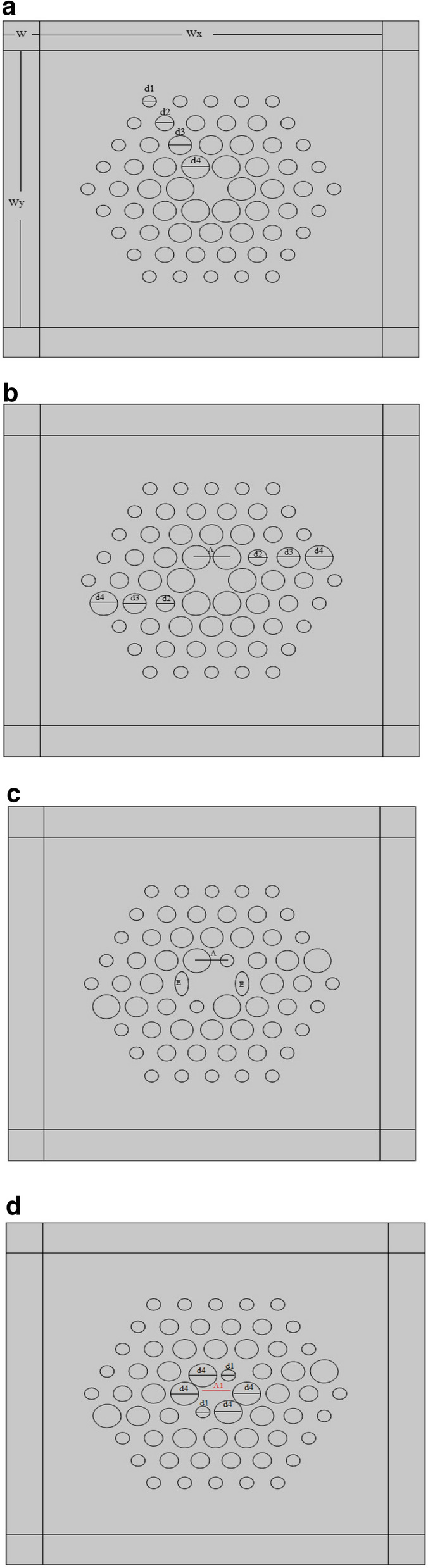
(**a**) PCF Structure, PCF 1. (**b**) PCF Structure, PCF 2. (**c**). PCF Structure, PCF 3. (**d**) PCF Structure , PCF 4.

**Figure 2:**
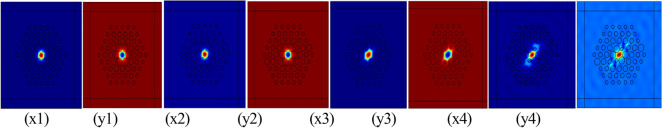
The fundamental mode field profile of x-polarisation and y-polarisation at 1.55 µm for; PCF1, PCF2, PCF3, PCF4 respectively.

**Figure 3:**
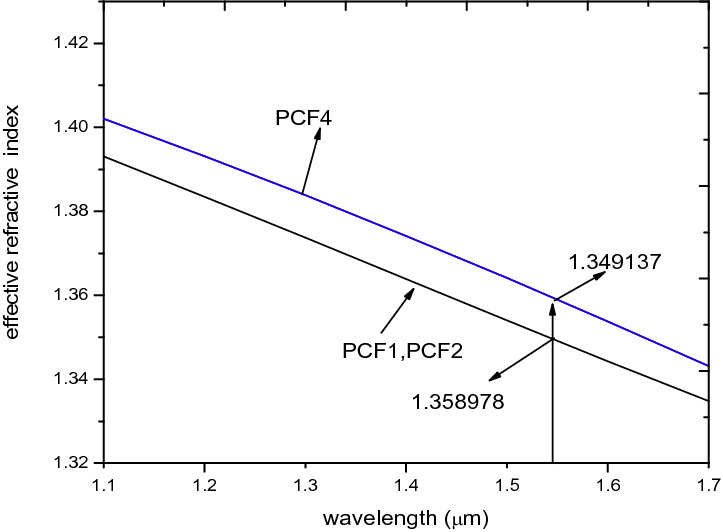
Effective refractive index of x-polarisation against wavelength for PCF1, PCF2, PCF3 and PCF4.

**Figure 4:**
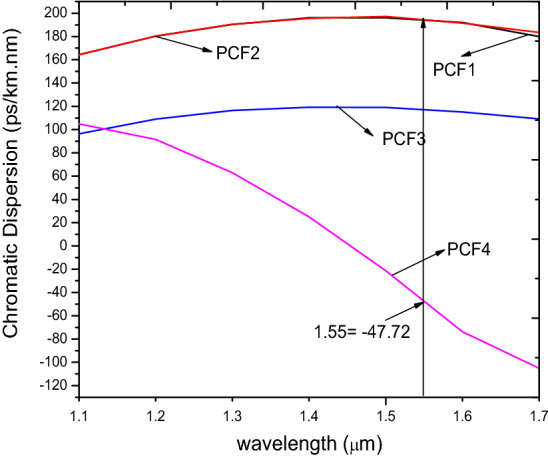
Chromatic dispersion of x-polarisation mode against wavelength for PCF1, PCF2, PCF3 and PCF4.

The proposed paper seeks to design and optimise a PCF structure that demonstrates high birefringence, negative chromatic dispersion and high nonlinearity. Four structures PCF1, PCF2, PCF3 and PCF4 which are all hexagonal with four air hole rings are presented in Figs. 1 to 4 respectively. Comsol multiphysics is used for the entire simulation. Simulation has been done in the range of wavelength of 0.7 µm to 2 µm.

PCF1 has been designed with air holes whose diameters increase along the main diagonal axis. The hole to hole spacing, ʌ is kept at 1.7 µm with an air filling fraction, d/ʌ fixed at 0.6. The air hole diameter (d) = 1.02 µm .The descending air holes from the outer ring to the inner air hole ring have been designed to follow the following pattern; d_1_ = 0.25d, d_2_ = 0.75d, d_3_ = d and d_4_ = 1.25d. The descending hole design is chosen due to its high nonlinearity and also most of the designs that is known to us did not consider it. The Perfectly matched layer (PML) has been designed with thickness of W = 2 µm, wx = 20.6 µm and wy = 24 µm. PCF1 is shown in Fig. 1a.

Figure 1b, shows PCF2 structure where the air hole sizes at the two orthogonal axes have been altered by arranging them in an ascending order in terms of diameter, whilst those at the inner ring are kept at d_4_ = 1.25d. The hole to hole spacing and air filling fraction are the same as that of PCF1.This PFC2 has been done to alter the symmetry of the structure in order to improve the birefringence.

In the case of PCF3 as shown in Fig. 1c, the birefringence is improved by two Elliptical holes E, of a-semi axes of 0.765 µm and b-semi axes of 1.53 µm which have been introduced into the inner ring. The other two air holes in the inner ring have been reduced to d_1_ = 0.25 but the last two remained as in PCF1. The pitch is varied in the order of 1.7 µm, 1.8 µm, 1.9 µm and 2.3 µm in order to determine the effect of the pitch on the optical properties in PCF3.

In PCF4, the elliptical holes have been replaced with circular ones. These two air holes are of diameter d_4_ whilst the rest are kept the same as in PCF3. However, the hole to hole spacing between the first and second ring is changed from ʌ = 1.7 to ʌ = 1.4. The full structure is shown in Fig. 1d.

The Fig. 2, shows the field profile of fundamental mode for all the PCF structures. The modal field distribution of the fundamental mode shows a well confined light in the core for PCF1,PCF2, PCF3 and some small leakage in PCF4. Comsol multiphysics is used for the simulation. Simulation has been done in the wavelength range of 0.7 µm to 2 µm.

## Results and discussion

This section investigates the effects of structural parameters such as pitch, hole sizes and air filling fraction on the performance parameters of the proposed PCF structures. Of specific interest are performance parameters such as birefringence, confinemet loss, chromatic dispersion and nonlinearity.

### The effect of Change of hole sizes in the two orthogonal axes

The effective refractive index decreases as the wavelength increase for x-polarisation as shown in Fig. 3 for PCF1 and PCF2. The graph indicates an insignificant change in the effective refractive index values of PCF1 and PCF2 which shows that the change in hole sizes at the two orthogonal axes do not significantly affect the effective refractive index.

A critical examination of the CD values reveal that they did not change as shown in Fig. 4 of PCF1 and PCF2 since the real effective index value determines the CD. This CD values agrees with the ascertion in^16^ that dispersion is affected by the holes in the inner ring since the field is propagated in the core.

**Figure 5:**
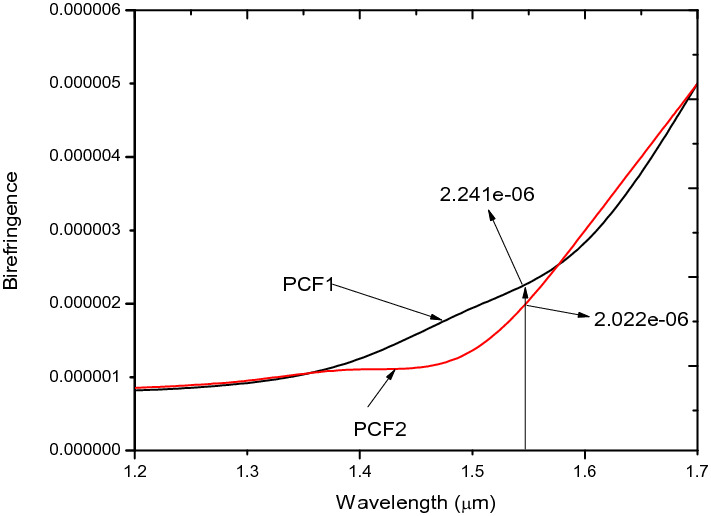
Birefringence against wavelength for PCF1, PCF2.

At 1.55 µm the B value in Fig. 5 for PCF1 is 2.241 × 10^–6^ and that of PCF2 is 2.022 × 10^–6^ which demonstrates that change in hole sizes at the two orthogonal axes of PCF2 did not affect B.

**Figure 6:**
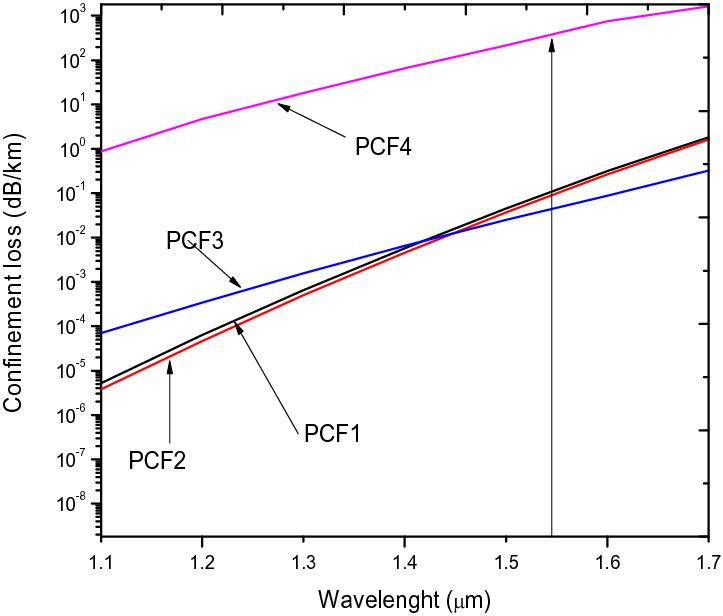
Confinement loss of x-polarisation against wavelength for PCF1, PCF2, PCF3 and PCF4.

The values of CL in Fig. 6 for PCF1 and PCF2 are 0.12654 dB/m and 0.104607 dB/m which is an indication of small reduction in leakage of light at the core in PCF1 as compared to PCF 2.

**Figure 7:**
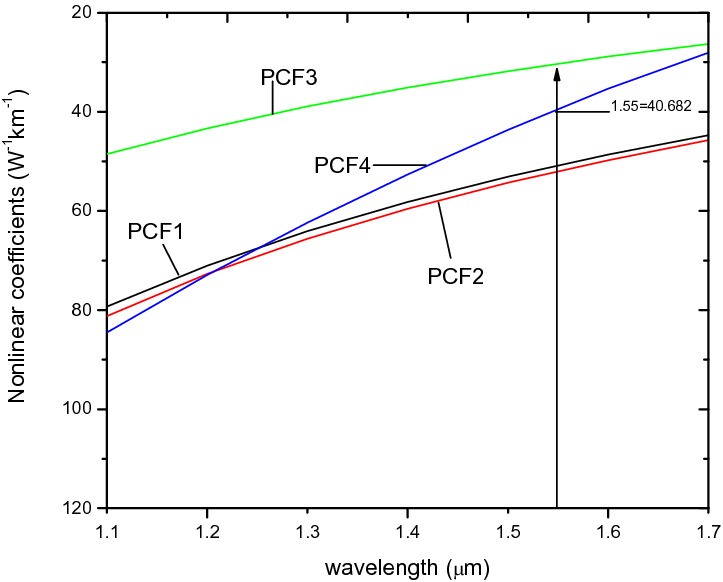
Nonlinear coefficients of x-polarisation against wavelength for PCF1, PCF2, PCF3 and PCF4.

The nonlinear coefficients in Fig. 7 for PCF1 and PCF2 are 52.445 W^−1^ km^−1^ and 52.446 W^−1^ km^−1^ respectively at 1.55 µm which indicates that the descending hole arrangement give rise to a very high nonlinearity.

### The effect of structural parameters of air holes in PCF 3

**Figure 8:**
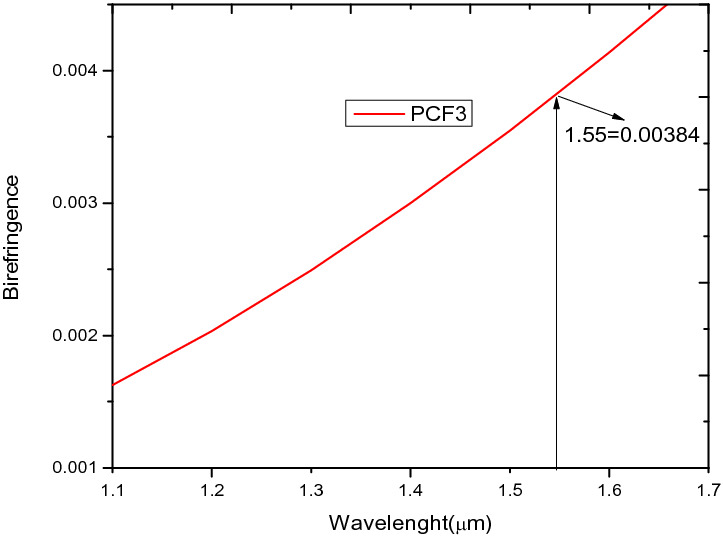
Birefringence against wavelength for PCF3.

**Figure 9:**
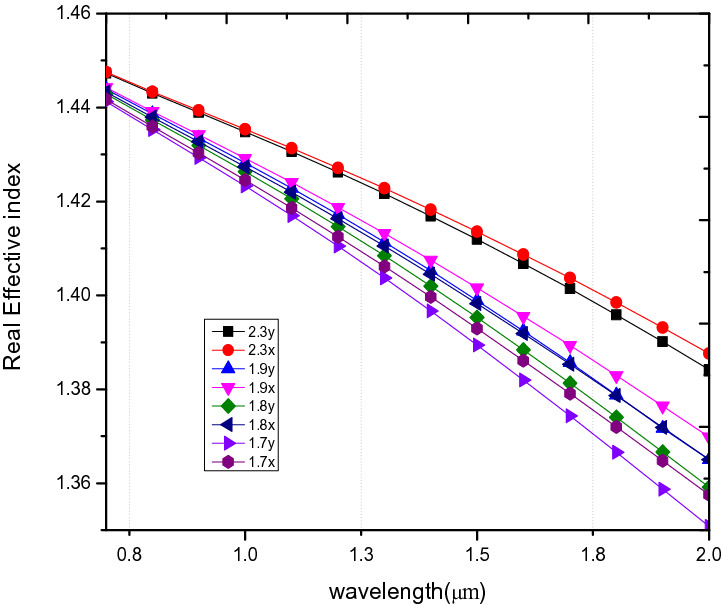
Variation of wavelength against real effective index, PCF3 for ʌ = 1.7 µm, 1.8 µm, 1.9 µm, 2.3 µm for x and y polarisation.

**Figure 10:**
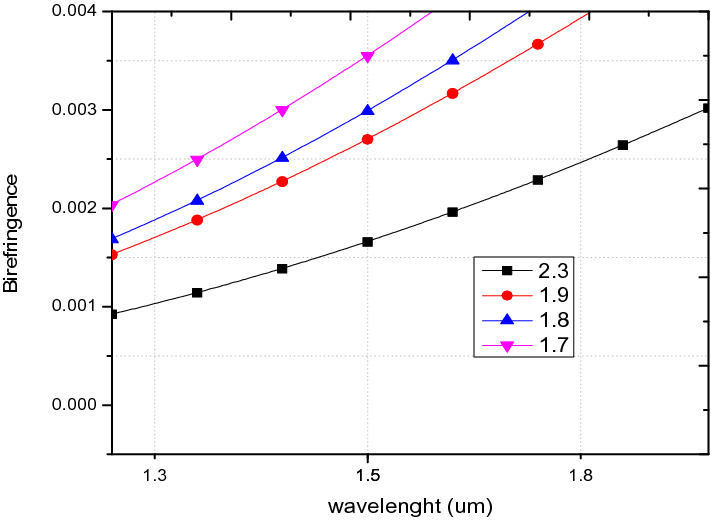
Variation of wavelength against birefringence for PCF3 at ʌ = 1.7 µm, 1.8 µm, 1.9 µm, 2.3 µm, d/ʌ = 0.6.

This section looks at the performance parameters of PCF3, which involves the introduction of elliptical air holes and the modification of d_4_ to d_1_. The introduction of elliptical air holes in the inner ring around the core in PCF3 increased the birefringence from 10^−6^ in Fig. 5 to 10^−3^ shown in Fig. 8, which is in agreement with work reported in^34,46^. However,The nonlinearity in Fig. 7, decreased from 52.445 W^−1^ km^−1^ for PCF 1 and PCF 2 to 31.32 W^−1^ km^−1^ for PCF 3 at 1.55 µm.

The CD as seen in Fig. 4, shows that both PCF1 and PCF2 possess flat profiles but that of PCF3 is relatively lower than both of them across the entire wavelength range.

The CL of PCF3 saw a steady decline to 0.04823 dB/m at 1.55 µm, which indicates a relatively better confinement of light in the core, compared to both PCF1 and PCF2 as shown in Fig. 6. Nonetheless, the change in the hole sizes at the orthogonal axes in PCF2 did not affect the CL and nonlinearity.

### Effect of Variation of the pitch in PCF3

Figure 9 shows a general decrease in the real effective refractive index as the wavelength increases for x polarisation. It can also be observed that as the hole to hole spacing (pitch) increases, the effective refractive index increase. The ʌ = 2.3 µm shows the highest values of the real effective index while that of ʌ = 1.7 µm indicates the lowest value. At 1.55 µm, the real effective index for ʌ = 1.7 µm, ʌ = 1.8 µm, ʌ = 1.9 µm, ʌ = 2.3 µm are 1.3896, 1.3951, 1.3986 and 1.4112 for x-polarisation.

The birefringence analysis shown in Fig. 10, shows that the highest value of birefringence recorded at 1.55 µm for the hole to hole spacing of 1.7 µm is 0.003838 which is in agreement with ^26,38,47–49^ and close to ^37,50^. The birefringence obtained for all pitch values are in the order of 10^–3^, which spans over a wavelength range of O to U optical communication bands.

**Figure 11:**
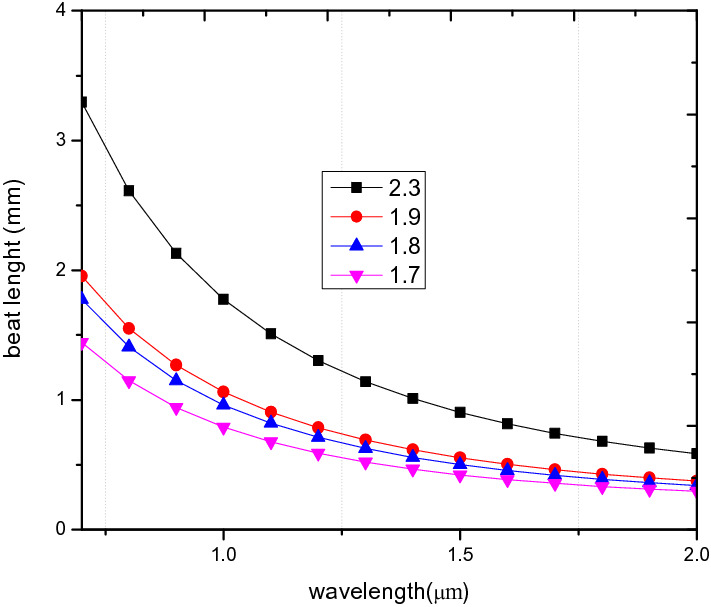
Variation of wavelength against beat length for PCF3 at ʌ = 1.7 µm, 1.8 µm, 1.9 µm, 2.3 µm, d/ʌ = 0.6.

Figure 11, indicates the relationship between the beat length and the wavelength. The plot shows that an in increase in ʌ results in an increase in the the beat length.

**Figure 12:**
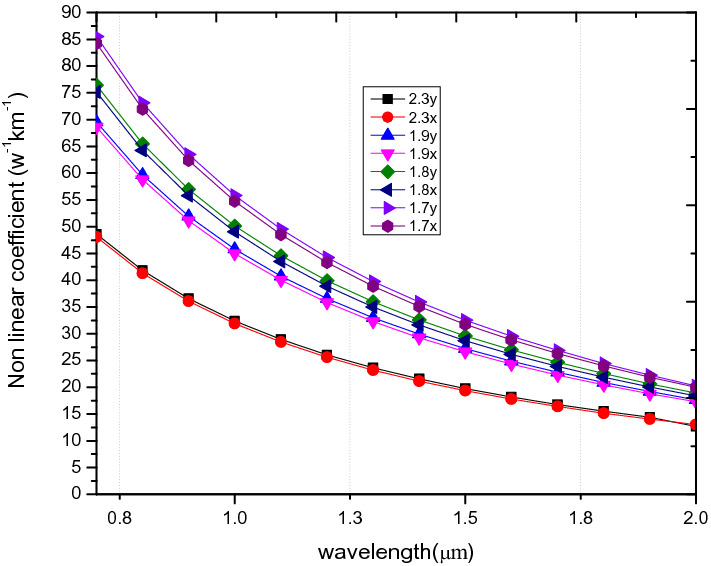
Variation of wavelength against nonlinear coefficient of PCF3 at ʌ = 1.7 µm, 1.8 µm, 1.9 µm, 2.3 µm, d/ʌ = 0.6.

Another performance parameter which is highly dependent on the pitch is the nonlinear coefficient. This is confirmend in Fig. 12, where it shown to be inversely propotional to the pitch and wavelength. Specifically, it is shown that a value of 31.3w^−1^ km^−1^ is obtained at a wavelength of 1.55 µm, for a pitch of 1.7 µm at x-polarization mode, whilst that for a pitch of 2.3 µm reduces to 19.158w^−1^ km^−1^ the same wavelength.

**Figure 13:**
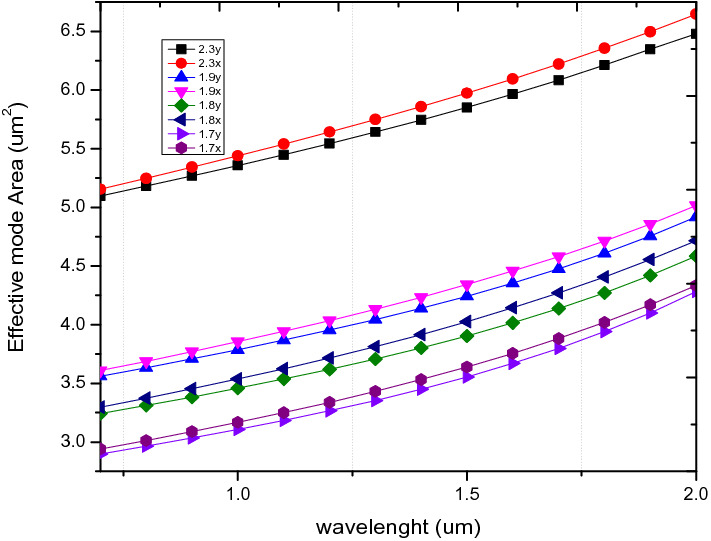
Variation of wavelength with effective mode area for ʌ = 1.7 µm, 1.8 µm, 1.9 µm, 2.3 µm, d/ʌ = 0.6.

Results from the analysis of the effective mode area which is shown in Fig. 13, shows that the effective mode area increases gradually with increase in wavelength. In relation to the pitch, the effective area increases with increase in pitch with the highest value at a pitch of 2.3 µm. The effective mode area at 1.55 µm for ʌ = 1.7 is 3.69µm^2^ for x-polarization mode which is close to ^51^ with d/ʌ = 0.6. These results indicate that unlike the nonlinear coefficient, the effective mode area is directly proportional to the pitch and wavelength.

**Figure 14:**
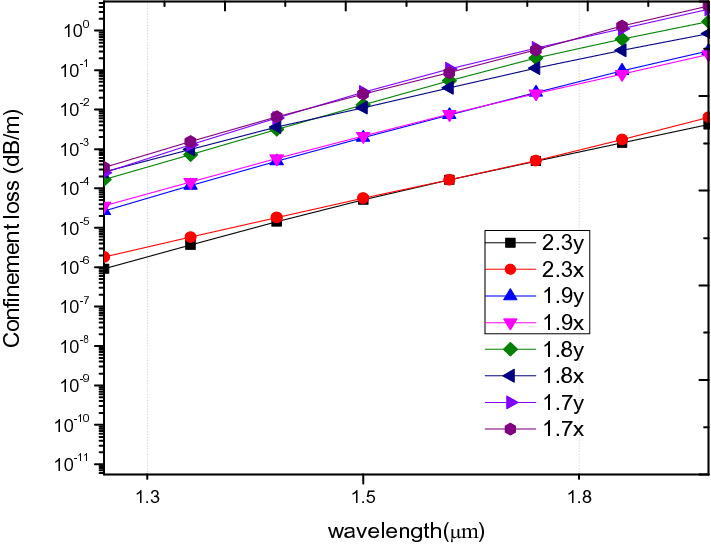
Variation of wavelength with confinement loss for ʌ = 1.7 µm, 1.8 µm, 1.9 µm, 2.3 µm, d/ʌ = 0.6.

In Fig. 14, CL decreases with increase in hole to hole spacing. The pitch, 2.3 µm at 1.55 µm indicates a confinement loss of 9.67 × 10^–5^ dB/m and 1.0034 × 10^–4^ for x and y polarization mode respectively which is lower than ^26,37,52^. The CL value shows that confinement loss can be improved by increasing the hole to hole spacing for a given air hole filling fraction.

**Figure 15:**
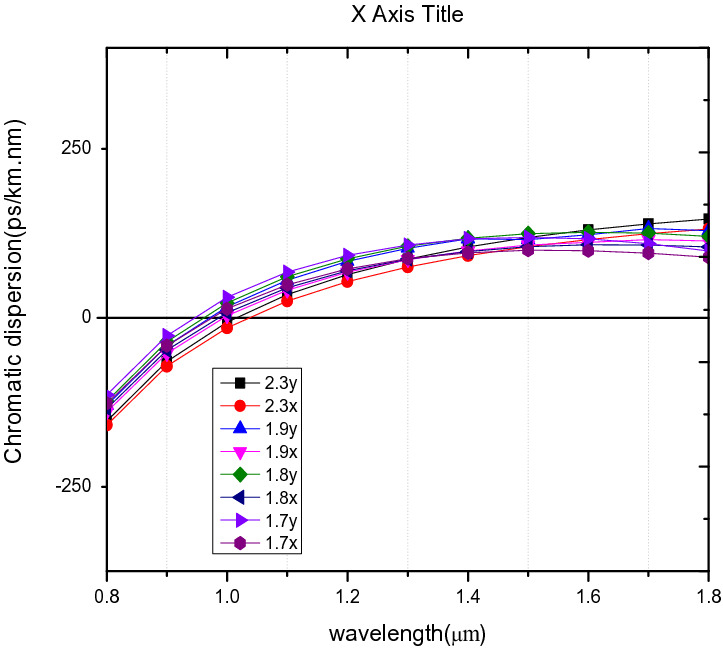
Variation of wavelength with chromatic dispersion (**a**) for ʌ = 1.7 µm, 1.8 µm, 1.9 µm, 2.3 µm, d/ʌ = 0.6.

The CD decreases as the pitch increases as shown in Fig. 15. Zero CD has been obtained for all the pitch values at a wavelength range of 0.9 µm to 1.1 µm similar to^53^. Positive dispersion is obtained for all the ʌ at 1.55 µm between 100 to 126 ps/nm/km.

### Performance characteristics of PCF4

The performance analysis presented on PCF 1–3 indicate the relative strengths and weaknesses of the respective structures. It gives an indication of the optimisation process, starting with PCF1 as the base structure and gruadually evolving it towards the optimised structure based on our desired performance parameters.

**Figure 16:**
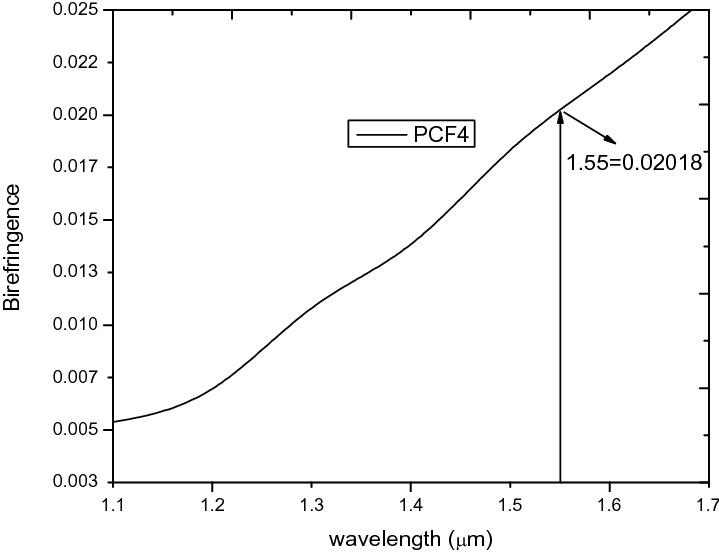
Birefringence against wavelength PCF4.

In this regard, a new structure called PCF4 as shown in Fig. 1d is derived from PCF 1–3. Comparing Fig. 8 to 16 shows that the B increases from 0.003838 in the case of PCF3 to 0.0202 for PCF4 at the same wavelength of 1.55 µm. This is higher that those reported in ^5,26,38^ and comparable to ^22,54^. The birefringence shown in Fig. 16, is in the order of 10^–2^ which covers the wavelength range from 1.3 µm to 1.7 µm. In conventional polarisation fibers it is reported that the modal birefringence is 5 × 10^–4^ ^28^ which is far lower than the B of both structures PCF3 and PCF4. The ultra-high birefringence achieved can be useful for applications in sensing and signal processing.

On the issue of nonlinearity, Fig. 7 shows that of PCF4 is 40.68 W^−1^ km^−1^, which is higher than 31.32 W^−1^ km^−1^ for PCF3 but lower than PCF1 and PCF2 at a wavelength of 1.55 µm. The nonlinearity value of 40.68 W^−1^ km^−1^ is higher than those reported in ^5,22,41,54^.

The chromatic dispersion for PCF 4 shown in Fig. 4 indicates a curvilinear shape declining towards the longer wavelengths. It has a zero dispersion point at the wavelength of 1.452 µm. The performance of the proposed structure, PCF4 of ultra high B is compared to other works as shown in Table 2.

**Table 2:** Comparison of the proposed PCF with other published works*.*

Study Reference	Structure	Nonlinearity [W^-1^ km^−1^]	Birefringence	Wavelength [µm]	Dispersion [ps/km.nm]
^5^	Circular and elliptical	39.330	2.82 × 10^–3^	1.3	−
^41^	All circular	26.7	−	1.3	+ 0.6402
^26^	Rhombic and circular	−	8 × 10^–3^	1.55	−
^38^	All Circular	−	1 × 10^–3^	1.55	− 20,186
^55^	Square	23.46	2.2 × 10^–3^	1.2	–
^54^	All circular	Below 20	10^–2^	1.55	–
^22^	Circular double cladding holes	24.48	3.11 × 10^–2^	1.55	− 513.6
Proposed PCF2	All circular	40.68	2.02 × 10^–2^	1.55	− 47.72

### Fabrication of proposed PCF

Fabrication of this structure is relatively easy as the number of air hole rings are only four and the air holes used are circular. There are several fabrication options such as extrusion^1^ sol–gel casting^2^ and drilling^3^. The conventional stack and draw technique^4^ has been used to fabricate PCF in^5^ which can also be used for the fabrication of this work. Furthermore, a PCF structure with a relatively smaller air filling fraction of 0.046 has been fabricated^6^, which means that our proposed PCF will be easier to fabricate. The proposed PCF can be fabricated using drilling, or two-step stack and draw method due to the use of circular holes^2,3,7,8^. The sol–gel fabrication method, which offers flexible design freedom with such a lattice structure and is robust against high amounts of bending can also be used for fabricating the proposed structure.

## Conclusion

The numerical analysis of a hexagonal four ring Photonic crystal fiber structure has been presented in this paper. It has been demonstrated that the proposed PCF structure with ultra-high birefringence of 2.02 × 10^–2^, negative chromatic dispersion of − 47.72 ps/km.nm and high nonlinearity of 40.68 W^−1^ km^−1^ at 1.55 µm for x-polarisation mode can be achieved. The proposed PCF can be fabricated using a two step stack and draw method or so gel casting method due to its relatively simple structure which consist of circular air holes. This structure with ultra high birefringence, high nonlinearity and negative CD would be a good candidate for optical fiber communication systems, polarisation maintaining and sensing applications.
