# Author Correction: Numerical analysis of photonic crystal fiber of ultra-high birefringence and high nonlinearity

**DOI:** 10.1038/s41598-021-96318-3

**Published:** 2021-08-17

**Authors:** Patrick Atsu Agbemabiese, Emmanuel Kofi Akowuah

**Affiliations:** Department of Computer Eng, Kwame Nkrumah University of Science and Technology, Kumasi, Ghana

Correction to: *Scientific Reports* 10.1038/s41598-020-77114-x, published online 03 December 2020

The original version of this Article contained errors in Table 1, where the parameters B1, B2 and B3 were incorrectly given as the parameters A1, A2 and A3. As a result, the parameters B1, B2 and B3 were repetitive.

The incorrect and correct sellmeier constants appear below.

Incorrect:

**Table:** 

Parameters	Constants
A1	0.69675
A2	0.408218
A3	0.890815
B1	0.047701
B2	0.0133777
B3	98.02107
C1	4.67914826e−3
C2	1.35120631e−2
C3	97.9340025

Correct:

**Table:** 

Parameters	Constants
B1	0.69675
B2	0.408218
B3	0.890815
C1	4.67914826e−3
C2	1.35120631e−2
C3	97.9340025

The original Article has been corrected.

